# Primary Versus Secondary Non-Urothelial Tumors Involving the Bladder: A 10-Year Analysis of Clinicopathologic Profiles and Adverse Feature Burden

**DOI:** 10.3390/cancers17203369

**Published:** 2025-10-18

**Authors:** Alexei Croitor, Vlad Dema, Alin Cumpanas, Razvan Bardan, Diana Herman, Mihail Nanu, Sorin Dema

**Affiliations:** 1 Department XV, Discipline of Urology, Faculty of Medicine, “Victor Babes” University of Medicine and Pharmacy, 300041 Timisoara, Romania; alexei.croitor@umft.ro (A.C.); cumpanas.alin@umft.ro (A.C.); razvan.bardan@umft.ro (R.B.); 2 Department of Pathology, “Pius Brinzeu” Clinical Emergency Hospital, 300723 Timisoara, Romania; diaherman@yahoo.com (D.H.);; 3 Oncology and Radiotherapy Department, Municipal Emergency Clinical Hospital, 300254 Timisoara, Romania; sorin.dema@umft.ro

**Keywords:** bladder neoplasms, non-urothelial carcinoma, adenocarcinoma, squamous cell carcinoma, neuroendocrine carcinoma, sarcomatoid urothelial carcinoma, lymph–vascular invasion, perineural invasion, surgical margins, secondary bladder involvement

## Abstract

**Simple Summary:**

Non-urothelial tumors can involve the bladder either as primary cancers (squamous, adenocarcinoma, neuroendocrine, and sarcomatoid) or as “secondary” spread from nearby organs (colon, prostate, and cervix). In a 10-year single-center cohort of 235 patients, secondary involvement was much more likely to show aggressive pathology than primary non-urothelial tumors, including deeper local invasion, tumors in vessels or nerves, and lymph-node metastases. Among secondaries, prostate origin had the highest burden of adverse features, followed by colorectal and cervical sources. These patterns support a practical approach: confirm tumor origin with modern immunohistochemistry, map the extent of disease carefully before surgery, and tailor the operation to obtain negative margins—favoring organ-sparing when anatomically feasible and escalating when margin risk is high (e.g., suspected prostate-to-trigone spread).

**Abstract:**

Background and Objectives: Non-urothelial bladder tumors and secondary bladder involvement from extravesical primaries are uncommon but clinically challenging. We compared clinicopathologic patterns between primary non-urothelial tumors and secondaries, and explored correlates of adverse pathologic features to inform diagnostic triage and surgical planning. Methods: We performed a single-center retrospective cohort (2014–2024) of consecutive bladder lesions meeting WHO 2022 criteria and AJCC 8th staging. Eligible cases were primary non-urothelial malignancies (squamous cell carcinoma (SCC), adenocarcinoma (ADK), small-cell/neuroendocrine (NEC), sarcomatoid) or secondary bladder involvement (colorectal, prostate, cervix, ovary, uterus, breast). Outcomes included advanced pT (≥pT3), lympho–vascular invasion (LVI), perineural invasion (PNI), nodal metastasis, margin status, and composite adverse events. Results: Of 235 analyzable cases, 59 were primary and 176 were secondary. Age and sex distributions were similar. Secondaries had a higher adverse burden: advanced pT 56.8% vs. 23.7%, LVI 47.2% vs. 27.1%, PNI 40.3% vs. 22.0%, node-positive 11.9% vs. 0%, and any adverse 65.3% vs. 33.9% (all significant). Histology composition differed (*p* < 10^−6^): secondaries were ADK-dominant (59.1%), whereas primaries were enriched for SCC (38.5%), sarcomatoid (28.8%), and NEC (21.2%). Among secondaries, prostate origin showed the most ominous profile (advanced pT 97.5%, PNI 77.5%, positive margins 64.7%); colorectal cases combined high advanced pT (70.2%) with lower margin positivity (27.6%). Adverse-feature count correlated with pT (ρ = 0.586). Conclusions: Secondary bladder involvement carries substantially higher adverse-pathology rates than primary non-urothelial tumors, with origin-specific risk gradients (prostate > colorectal ≳ cervix). Rigorous origin adjudication and a margin-focused, anatomy-adapted surgical strategy may improve outcomes; prospective outcome-linked validation is warranted.

## 1. Introduction

Non-urothelial tumors involving the urinary bladder encompass a diverse group of primary epithelial and non-epithelial malignancies (primary squamous cell carcinoma, adenocarcinoma, small-cell/neuroendocrine, and sarcomatoid variants) as well as secondary involvement by extravesical primaries via direct extension or metastasis. Contemporary classification aligns entities by lineage and molecular features, refining diagnostic criteria and variant recognition in ways that materially affect staging, prognosis, and treatment selection [1,2]. International clinical guidelines situate these rare phenotypes within broader management pathways that remain dominated by urothelial carcinoma evidence, underscoring the need for focused data on non-urothelial diseases and on secondary bladder involvement [3,4].

Urothelial carcinoma accounts for approximately 90% of bladder cancers in industrialized settings; primary non-urothelial histologies such as squamous cell carcinoma, adenocarcinoma (urachal and non-urachal), small-cell/neuroendocrine, and sarcomatoid variants are uncommon but frequently present with adverse pathology and worse outcomes than conventional urothelial tumors [5,6,7,8,9,10,11,12]. Secondary bladder involvement arises via direct extension from adjacent organs (notably the colorectum, prostate, cervix/uterus/ovary) or, less commonly, by metastasis (breast, lung, melanoma) and can mimic primary disease clinically and endoscopically [13,14,15].

Accurate origin adjudication—integrating imaging, operative findings, and immunohistochemistry (CK7/CK20 patterns, β-catenin localization, SATB2/cadherin-17 for colorectal, NKX3.1/PSA for prostate, and GATA3 for urothelial lineage)—is essential because staging, resection strategy, and adjuvant pathways diverge meaningfully by source [16,17,18,19,20]. We therefore compared clinicopathologic features between primary non-urothelial tumors and secondary bladder involvement in a consecutive 10-year cohort and quantified the distribution of adverse pathologic features by origin to inform diagnostic triage and margin-focused surgical planning.

## 2. Materials and Methods

### 2.1. Study Design, Setting, and Ethical Oversight

We performed a single-center, retrospective observational cohort study at a tertiary academic network comprising the university-affiliated Department of Urology, Pathology, and Oncology in Timișoara, Romania. Consecutive bladder tumor resections and biopsies logged in the institutional pathology archive between 1 January 2014 and 31 December 2024 were screened. The study adhered to the Declaration of Helsinki and EU-GDPR. The institutional ethics committee approved secondary analysis of de-identified data with a consent waiver for archival material. Data were stored on an encrypted file; only authorized investigators accessed the dataset.

### 2.2. Case Identification and Eligibility Criteria

Eligible cases met all of the following: (i) a bladder lesion sampled by TURBT, partial cystectomy, radical cystectomy, en bloc multivisceral resection, or image-guided biopsy; (ii) a non-urothelial phenotype on final pathology (primary squamous cell carcinoma [SCC], primary adenocarcinoma [ADK: urachal or non-urachal], small-cell/neuroendocrine carcinoma [NEC], or sarcomatoid urothelial carcinoma); or secondary bladder involvement by an extra-vesical primary (colorectal, prostate, cervix, ovary, uterus, breast); and (iii) sufficient histology and clinicopathologic data to adjudicate adverse features and margin status.

We excluded the following: (i) pure conventional urothelial carcinoma (including micropapillary, plasmacytoid, nested, and other urothelial variants without sarcomatoid transformation); (ii) non-neoplastic mimics; (iii) cases with indeterminate origin after full clinicopathologic work-up; and (iv) duplicate submissions from the same operative event (the most definitive resection was retained).

### 2.3. Pathology Review, Ancillary Testing, and Origin Adjudication

Histotypes were assigned using WHO 2022 criteria [21]. Tumors were staged according to AJCC 8th edition TNM [22]. When the origin was uncertain, we integrated imaging, endoscopy, operative notes, and immunohistochemistry (IHC). Secondary involvement required either: (i) a proven extravesical primary with contiguous invasion on imaging/operative report and concordant IHC or (ii) a metastatic phenotype with IHC/clinical course favoring an extravesical origin.

For adverse features (LVI, PNI), reporting followed routine departmental criteria on H&E with adjunct endothelial markers when needed; however, we did not institute a separate blinded re-review across all cases, which may introduce interobserver variability. Discrepancies were resolved by consensus.

### 2.4. Variables, Definitions, and Data Quality

We recorded age (years), sex, and calendar year of diagnosis. Age distributions are summarized by mean ± SD and median with interquartile range (IQR) for comparability with prior series.

Histology categories for analysis matched ADK, SCC, sarcomatoid, NEC, and “other” (melanoma, lymphoma-rare). For exploratory analyses within primaries, we defined “aggressive primaries” as NEC or sarcomatoid; all other primary non-urothelial histologies were grouped as “non-aggressive.” Tumor grade used ordinal coding (GradeNum) aligned to site-specific systems (low/high for SCC and ADK; high-grade assumed for NEC and sarcomatoid).

Pathologic T category (pTbase) and nodal status (pN) followed AJCC 8th. Advanced T was defined a priori as pT ≥ 3. The adverse-feature framework mirrored your tables:

- Node+: pN ≥ 1;
- LVI: unequivocal intravascular tumor emboli on H&E ± D2-40/CD31 as available;
- PNI: tumor within any of the three perineural compartments;
- Margin positivity: R1 (microscopic) or R2 (macroscopic).

A composite endpoint, any adverse, was coded present if ≥1 of Node+, Advanced T, LVI, PNI, or R1/R2 was present. When a feature was unassessable (LVI in fragmented TURBT), denominators (*n*/N) reflect available assessments. The composite was intended as a pragmatic triage summary; given the heterogeneity of its components, all individual features are also reported and interpreted separately, and clinical decisions should prioritize component-level detail.

Margin cohorts were R0 (negative) vs. R1/R2 (positive). Secondary sites were abstracted verbatim from the primary tumor diagnosis (colon, prostate, cervix, ovary, uterus, breast) and analyzed both individually and as colorectal vs. non-colorectal.

The primary comparative outcome was the distribution of adverse pathologic features between primary and secondary bladder tumors. Secondary outcomes included the following: (i) histology composition by origin; (ii) correlations among age, grade, stage, and adverse-feature count; (iii) subgroup contrasts (aggressive vs. non-aggressive primaries; colon vs. prostate vs. cervix secondaries; colorectal vs. non-colorectal secondaries); and (iv) margin-status cohorts across all origins.

### 2.5. Statistical Analysis

Continuous variables were summarized as mean ± SD, median (Q1–Q3), and range; normality was screened with the Shapiro–Wilk test. Between-group comparisons used the Mann–Whitney U test for continuous data and Pearson’s χ^2^ or Fisher’s exact test for categorical data. Effect sizes are reported as rank-biserial correlation (Mann–Whitney) and Cramer’s V (χ^2^). For key proportions, 95% CIs used Wilson intervals.

Global composition differences (histology by tumor origin) were evaluated with omnibus χ^2^ tests followed, when significant, by Holm-adjusted pairwise column-proportion tests. Monotone associations were quantified with Spearman’s ρ. Prespecified subgroup analyses included, among primary tumors, aggressive (neuroendocrine/sarcomatoid) versus non-aggressive categories; among secondary tumors, colon versus prostate versus cervix (global χ^2^ with Holm-adjusted pairwise contrasts), plus colorectal versus non-colorectal comparisons for each adverse feature and the composite burden. Margin status was compared across R0 versus R1/R2 resections using χ^2^ tests.

For multivariable inference, a logistic regression modeled “Any adverse” (yes/no) with covariates: primary vs. secondary tumor, age, aggressive histology, numeric grade, and sex. Results are reported as odds ratios with 95% CIs from exponentiated coefficients. Complete-case analysis was used; no penalization was applied, and multicollinearity was low (all VIFs < 2). Pairwise tests prompted by significant omnibus tests were Holm-adjusted; all other tests were two-sided with α = 0.05. Analyses were conducted in R v4.3.

Given limited event counts, subgroup and multivariable analyses were treated as exploratory and hypothesis-generating; primary inferences relied on prespecified univariable contrasts with multiplicity control where applicable. This was a convenience cohort spanning 11 years. Feasibility estimates indicated that detecting a ≥20-percentage-point difference in “Any adverse” between origins at α = 0.05 with 80% power required ~180 secondaries and ~55 primaries; the analytic sample (secondary *n* = 176; primary *n* = 59) was close to this target, supporting adequate precision for the primary outcomes.

## 3. Results

Secondary tumors (*n* = 176) and primary tumors (*n* = 59) presented at a similar age (median 68 vs. 68 years; Mann–Whitney *p* = 0.662), with broadly overlapping dispersion (Q1–Q3: 60–74.2 vs. 61–75). Sex distribution also did not differ significantly (female 42.6% vs. 54.2%; χ^2^ *p* = 0.161), as seen in Table 1.

Secondary tumors showed a substantially higher adverse feature load than primaries, including advanced pathological stage (pT ≥ 3: 56.8% vs. 23.7%; *p* = 2.1 × 10^−5^), LVI (47.2% vs. 27.1%; *p* = 0.0109), and PNI (40.3% vs. 22.0%; *p* = 0.0172). Node positivity occurred only among secondaries (11.9% vs. 0%; *p* = 0.0118). Margin positivity rates were similar where available (≈50%), and the composite “any adverse” metric was nearly doubled in secondaries (65.3% vs. 33.9%; *p* = 4.6 × 10^−5^), as presented in Table 2.

Histologic composition differed profoundly by origin (global χ^2^ *p* < 1 × 10^−6^): secondaries were dominated by adenocarcinoma (ADK 59.1%), reflecting common colorectal/gynecologic sources, with fewer SCC (22.8%), sarcomatoid (8.8%), and NEC (6.4%). Primaries, by contrast, were enriched for SCC (38.5%), sarcomatoid (28.8%), and NEC (21.2%) and contained far fewer ADK (15.4%), as described in Table 3.

Adverse burden correlated strongly with baseline pT (ρ = 0.586; *p* = 3.17 × 10^−16^), indicating that higher T category co-occurs with accumulation of adverse features. Age showed weak, negative correlations with both pT (ρ = −0.179; *p* = 0.028) and total adverse count (ρ = −0.140; *p* = 0.032), while grade had no meaningful association with adverse count (ρ = −0.018; *p* = 0.878), suggesting stage, rather than age or grade, is associated with risk accumulation (Table 4).

Across secondary sources, disease severity varied: prostate involvement showed near-universal advanced T (97.5%) and the highest PNI (77.5%) and margin positivity (64.7% where assessed), while colorectal involvement had high advanced T (70.2%) with intermediate PNI (38.3%) and lower margin positivity (27.6%). Cervical sources exhibited moderate-to-high rates across features (e.g., LVI 52.6%), and rarer sites (ovary, uterus, and breast) were too small for stable estimates; age distributions were broadly similar across groups (Table 5).

Aggressive primaries trended toward greater LVI (38.5% vs. 18.2%) and higher composite adverse burden (46.2% vs. 24.2%), though differences did not reach statistical significance in this cohort. Advanced T occurred with similar frequency (≈23–24%), and nodal metastasis was absent in both groups (Table 6).

Prostate secondaries exhibited the most ominous profile (advanced T 97.5%, PNI 77.5%, any adverse 100%), significantly exceeding colon and cervix for several endpoints (global χ^2^ *p* ≤ 0.0007). Colorectal secondaries showed high advanced T (70.2%) and LVI (61.7%) with intermediate PNI (38.3%), while cervical secondaries were comparatively lower on several metrics but still demonstrated substantial adverse rates (e.g., any adverse 68.4%). Margin positivity was highest in prostate (64.7%) versus colon (27.6%) and cervix (50%), as described in Table 7.

Exploratory multivariable models did not identify stable independent associations beyond origin, consistent with limited events; effect-size estimates are therefore not emphasized and are omitted from the main text.

Within primaries, sarcomatoid tumors showed the highest adverse burden (advanced T 40.0%, any adverse 46.7%) and frequent LVI/PNI (each 33.3%), whereas NECs displayed intermediate risks (any adverse 36.4%) and “other primaries” the lowest (any adverse 27.3%). Nodal metastasis was absent across primary subtypes in this series (Table 8).

Compared with non-colorectal secondaries, colorectal cases carried higher advanced T (70.2% vs. 51.9%; *p* = 0.046) and LVI (61.7% vs. 41.9%; *p* = 0.031), and a greater composite adverse rate (78.7% vs. 60.5%; *p* = 0.038). Margin positivity was paradoxically lower in colorectal (27.6% vs. 60.0%; *p* = 0.015), perhaps reflecting different operative planes and resection strategies in multivisceral surgery; nodal involvement did not differ significantly (19.1% vs. 9.3%; *p* = 0.129), as described in Table 9.

Cases with positive margins (R1/R2) exhibited uniformly high adverse profiles (any adverse 100%) and slightly more frequent LVI (79.5% vs. 70.5%) and PNI (66.7% vs. 75.0%) compared with R0 resections; however, none of the between-group differences reached statistical significance, likely due to small denominators and selection effects (Table 10).

**Figure 1:**
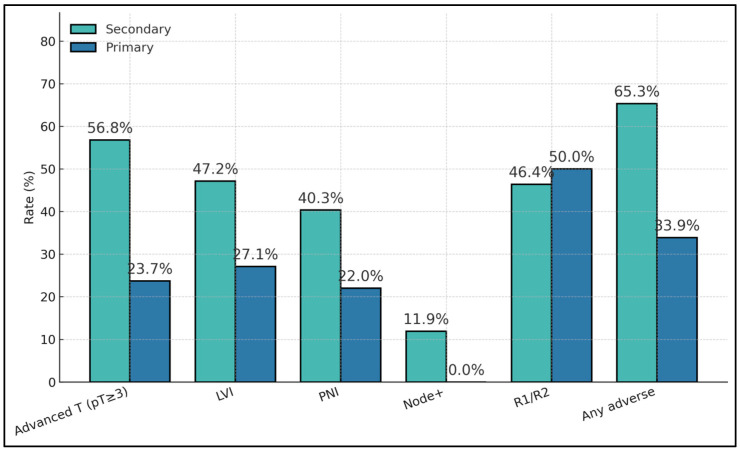
Adverse feature rates by origin.

Secondary bladder tumors showed consistently higher adverse-pathology rates than primaries: advanced T (pT ≥ 3) 56.8% vs. 23.7%, LVI 47.2% vs. 27.1%, PNI 40.3% vs. 22.0%, and any adverse 65.3% vs. 33.9%. Nodal positivity was present in secondaries (11.9%) but absent in primaries (0%), reinforcing more advanced regional spread among metastatic cases. Margin positivity (R1/R2) was comparable (46.4% vs. 50.0%), suggesting resection status is influenced by local technical/anatomic factors rather than origin alone (Figure 1).

**Figure 2:**
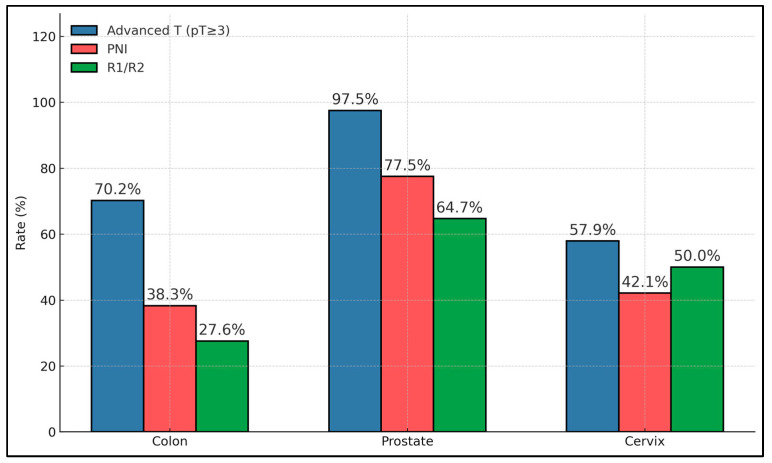
Secondary subgroup comparison.

Within secondary tumors, prostate metastases were the most aggressive by pathology, with advanced T 97.5%, PNI at 77.5%, and R1/R2 at 64.7%. Colon secondaries had high advanced T (70.2%) but more favorable margins (R1/R2 at 27.6%) and lower PNI (38.3%). Cervix secondaries showed intermediate aggressiveness (advanced T 57.9%, PNI 42.1%) yet relatively high margin positivity (50.0%). Overall, the site of origin among secondaries meaningfully stratifies risk features, with prostate >> colon ≳ cervix for combined adverse pathology burden (Figure 2).

## 4. Discussion

Our observation that secondary bladder involvement carries a heavier burden of adverse features than primary non-urothelial primaries is consistent with contemporary colorectal–urologic series showing that direct extension into the bladder typically reflects advanced locoregional biology and surgically challenging planes. In a multi-decade cohort comparing en bloc partial versus total cystectomy for colorectal primaries, Kondo et al. reported that patients selected for total cystectomy had longer operations, more complications, and worse oncologic outcomes than those undergoing partial cystectomy—an indirect marker of locally aggressive disease at presentation [23].

Survival-oriented datasets focusing specifically on secondary bladder tumors likewise underscore the gravity of such diagnoses; Hamza et al. found mean survival after recognition of bladder metastasis of ~18 months across heterogeneous primaries, aligning with our “adverse feature clustering” in secondaries [24]. More recent single-center colorectal series also support that complete en bloc resection can be curative in selected cases, but case selection hinges on avoiding trigonal/neck involvement and securing negative margins, which are harder to achieve when secondary spread is bulky or perineural/lympho–vascular routes are dominant [25]. These converging data explain why our secondary group concentrated on advanced pT, LVI, PNI, and nodal positivity.

Within secondaries, our cohort’s most ominous composite profile in prostate-origin cases—near-universal advanced pT, very high PNI, and the highest margin positivity—fits the well-described perineural tropism of prostate carcinoma. A meta-analysis of localized disease (surgery or radiotherapy) showed that PNI is independently associated with higher biochemical recurrence risk [26], and broader syntheses confirm PNI as a credible adverse prognostic factor in prostate cancer biology and a potential signal to consider treatment intensification [27,28]. Mechanistically, perineural tracks facilitate tumor spread from the prostate base toward the bladder neck and trigone; in surgical series, this anatomic corridor correlates with more complex dissections and an increased probability of positive margins. Our results—PNI ~78% and R1/R2 ~65% in prostatic secondaries—are therefore biologically coherent with the literature and emphasize preoperative attention to neurovascular bundle involvement on imaging and endoscopy. Practically, when PNI-rich prostatic invasion is suspected, surgical planning should prioritize wider en bloc planes and vigilant intraoperative margin control to offset the margin risk that we and others observe.

The colorectal subgroup in our data exhibited higher rates of advanced pT and LVI and a higher composite adverse endpoint than non-colorectal secondaries, yet a lower margin positivity rate. Several lines of evidence reconcile this apparent paradox. First, comparative cohorts indicate that partial cystectomy is often feasible for dome/anterior wall invasion, with acceptable long-term survival when margins are negative, whereas total cystectomy tends to be reserved for trigone/neck involvement or extensive adherence—situations that portend worse pathology and outcomes [23]. Second, independent surgical series show that en bloc partial cystectomy can achieve R0 with preserved function in well-selected patients, while total cystectomy carries higher morbidity without guaranteed oncologic advantage in all settings [23,25]. Third, recent studies and contemporary institutional experiences emphasize meticulous preoperative mapping (contrast CT/MRI, cystoscopy) to discriminate true invasion from inflammatory adhesions, thereby avoiding overtreatment and enabling organ-sparing resections when oncologically sound [29]. Moreover, the composite “Any adverse” tracked closely with pT, but component-level differences (e.g., PNI in prostate-origin cases vs. margin status in colorectal) better reflect mechanism and should guide management.

Our primary cohort’s enrichment for sarcomatoid and small-cell histologies echoes the literature in which both variants are uncommon but lethal. For sarcomatoid urothelial carcinoma, multicenter analyses show limited response to neoadjuvant chemotherapy and inferior outcomes versus conventional urothelial carcinoma even after radical cystectomy [30]. Broader, cross-subtype urothelial work further highlights the aggressive genomic and clinical phenotype of sarcomatoid tumors, supporting early systemic therapy and radical local control when feasible [31]. Small-cell carcinoma of the bladder follows a similarly unfavorable course: the largest UK consortium reported poor overall survival across stages but better outcomes for organ-confined disease and for patients able to receive platinum chemotherapy, aligning with lung small-cell treatment paradigms [32]. More recent contemporary series and reviews maintain that multimodal therapy (platinum doublets with definitive surgery or chemoradiation) remains the cornerstone for early-stage disease, with systemic therapy essential in advanced settings [33]. These external data substantiate our internal pattern: even where pT proportions appear similar to other primaries, aggressive histology accumulates LVI/PNI and composite adverse features, warranting intensification beyond urothelial “defaults.”

The strong correlation we observed between pT and cumulative adverse features is expected, but our data also show substantial rates of LVI/PNI—features that add prognostic information beyond stage. Multiple meta-analyses in radical cystectomy populations identify LVI as an independent predictor of recurrence and cancer-specific mortality, with the effect most pronounced in node-negative patients (where it can “phenocopy” nodal involvement) [34,35]. Classic cystectomy pathology studies also flagged both vascular and perineural invasion as independent adverse factors for survival, a result repeatedly re-demonstrated in modern cohorts [36]. Extrapolation from the upper-tract literature, where LVI is likewise a robust adverse marker, reinforces biological plausibility and the value of standardized reporting [34]. Taken together, these data argue that routine, high-quality assessment of LVI/PNI should influence adjuvant therapy decisions and surveillance intensity—particularly relevant in our series where some nodal sampling was limited and where the “any-adverse” composite strongly tracked with pT. Incorporating LVI/PNI into risk tools may improve discrimination and clinical decision-making beyond stage alone.

Because our secondary group is adenocarcinoma-dominant—reflecting colorectal/gynecologic sources—diagnostic adjudication is pivotal. Beyond CK7/CK20, several studies support SATB2 and cadherin-17 (CDH17) as highly sensitive and specific colorectal markers, including in poorly differentiated tumors; their combined use improves accuracy over older two-marker panels and helps avoid misclassification of primary bladder adenocarcinoma [37,38]. Complementary work demonstrates the utility of β-catenin localization (nuclear in colorectal, membranous in primary bladder) and emphasizes CDH17/SATB2 when morphology is equivocal or when lesions arise at the dome/anterior wall where urachal versus colorectal differentials are common [39]. Even in cytology, adding SATB2/CDH17 enhances colorectal assignment versus standard GI panels, supporting reflex testing in limited material [40]. Operationally, our findings endorse a reflex algorithm: SATB2 ± CDH17 (with CDX2 as needed), assessment of β-catenin localization, and site-specific markers (e.g., NKX3.1 for prostate) to secure origin before definitive surgery. Such rigor reduces staging ambiguity and prevents inflation of “primary adenocarcinoma” tallies that could distort adverse-feature benchmarking.

Our R1/R2 subgroup—unsurprisingly—co-segregated with universal composite adverse status, reiterating that margin negativity dominates long-term control when the bladder is involved by adjacent organ cancers. Contemporary colorectal series emphasize that R0 resection is the pivotal variable; when anatomically feasible, partial cystectomy yields oncologically acceptable outcomes with better functional preservation, while total cystectomy is reserved for trigone/neck or diffuse involvement and carries higher morbidity [23,25]. Recent reviews stress the importance of multidisciplinary mapping (MRI for trigone/neck, cystoscopy for mucosal breach) to avoid over-calling invasion and to plan precise en bloc planes; when preoperative suspicion for extensive perineural spread (as in prostate) or multifocal adverse features exists, teams should lower the threshold for more radical resection to secure margins [41]. In short, our data back a pragmatic algorithm: adjudicate origin with modern IHC, stage meticulously, and pursue the least morbid operation compatible with R0—escalating when anatomy or biology (e.g., PNI-rich trajectories) makes margin compromise likely.

While our dataset lacked longitudinal outcomes, multiple external cohorts inform prognosis. Population-level analyses indicate that patients with secondary or radiation-associated bladder cancers undergoing radical cystectomy experience cancer-specific mortality comparable to primary bladder cancer when matched by stage, though other-cause mortality can be higher in selected subgroups [42]. Dedicated series of secondary bladder involvement also report limited median survival following recognition of bladder disease, underscoring the adverse biology captured by our clustering of advanced pT, LVI/PNI, and nodal spread [24]. For colorectal primaries with pathologic bladder invasion, contemporary cohorts suggest that, when anatomically appropriate, partial cystectomy within en bloc resections can achieve negative margins with favorable functional and oncologic outcomes compared with total cystectomy [23,25,29]. These external results are concordant with our origin-specific pathology gradient (prostate > colorectal ≳ cervix) and with the primacy of achieving R0 resection.

Population-based analyses of variant-histology bladder cancer treated with radical cystectomy report that female sex is associated with higher rates of non-organ-confined disease and worse cancer-specific mortality [43]. In our series, sex distribution did not differ significantly between origin groups and was not independently informative in exploratory models; however, the study was not powered for sex-stratified effects within histologies. Future outcome-linked work should examine whether sex modifies the relationship between origin, adverse features, and prognosis in non-urothelial/secondary disease.

The retrospective archive lacked standardized linkage to recurrence or survival endpoints across the full study interval, preventing robust time-to-event analyses. Consequently, our comparisons emphasize clinicopathologic risk features rather than outcomes, and should be validated prospectively with outcome capture. Histology- and site-specific denominators were small for rarer subgroups (ovary, uterus, breast), reducing power and precision for between-group comparisons. Margin assessments and nodal staging were incomplete in a subset, which can inflate composite “any adverse” rates or obscure true effect sizes. Centralized pathology re-review was not performed for all cases, so interobserver variability in LVI/PNI reporting and pT assignment is possible. We did not have reliable survival or recurrence capture across the study period, precluding time-to-event analyses. Accordingly, our inferences emphasize pathology-based risk; outcome-linked validation in a prospective, registry-enabled cohort is warranted. We did not perform centralized blinded re-review of LVI/PNI beyond routine consensus for discrepant cases; modest misclassification is possible. Finally, we lacked standardized preoperative imaging adjudication and longitudinal outcomes (recurrence, survival), precluding evaluation of how adverse features translated into prognosis and limiting causal inferences.

## 5. Conclusions

Secondary bladder involvement carried a substantially heavier adverse feature burden than primary non-urothelial primaries, with prostate-derived cases displaying the most ominous profile (notably high PNI and margin positivity). Colorectal secondaries combined advanced pT/LVI with comparatively lower margin positivity, consistent with the feasibility of precise en bloc partial cystectomy in selected anatomy. Among primaries, sarcomatoid, followed by small-cell, showed the greatest adverse accumulation. LVI and PNI emerged as cross-cutting risk anchors beyond T-stage. Clinically, rigorous origin adjudication with modern IHC panels, meticulous preoperative mapping, and a margin-focused operative strategy that favors organ-sparing when R0 is achievable and escalates when trigone/neck or PNI-rich spread is suspected, may optimize outcomes.

## Figures and Tables

**Table 1:** Baseline characteristics of patients by origin.

Origin	*n*	Age Mean	SD	Median	Q1	Q3	Min	Max	Female *n*	Female %
Secondary	176	67	10.1	68	60	74.2	36	90	75	42.6
Primary	59	67.8	9.4	68	61	75	50	90	32	54.2

Group tests: Age (Mann–Whitney U) = 0.661868; Sex (χ^2^) = 0.161365; SD—standard deviation; Q1/Q3—first/third quartile; χ^2^—chi-square test.

**Table 2:** Pathology by origin.

Variable	Primary n/N	Primary %	Secondary n/N	Secondary %	*p*-Value (χ^2^)
Advanced T (pT ≥ 3)	14/59	23.7	100/176	56.8	0.000021
Node-positive (pN ≥ 1)	0/59	0	21/176	11.9	0.011845
Lymphovascular invasion (LVI)	16/59	27.1	83/176	47.2	0.010908
Perineural invasion (PNI)	13/59	22	71/176	40.3	0.017205
Positive margin (R1/R2)	7/14	50	32/69	46.4	1
Any adverse (composite *)	20/59	33.9	115/176	65.3	0.000046

* Any adverse = any of: Node+, Advanced T, LVI, PNI, or R1/R2; pT—pathologic T stage; pN—pathologic nodal stage; LVI—lympho–vascular invasion; PNI—perineural invasion; R1/R2—microscopically/macroscopically positive surgical margin; χ^2^—chi-square test.

**Table 3:** Histology by origin (counts with column %).

Histology Category	Secondary (*n*, %)	Primary (*n*, %)
ADK	101 (59.1%)	8 (15.4%)
SCC	39 (22.8%)	20 (38.5%)
Sarcomatoid	15 (8.8%)	15 (28.8%)
NEC	11 (6.4%)	11 (21.2%)
Other	2 (1.2%)	1 (1.9%)

Global χ^2^ across full histology table: *p* < 1 × 10^−6^ (significant composition differences); ADK—adenocarcinoma; SCC—squamous cell carcinoma; NEC—neuroendocrine carcinoma; χ^2^—chi-square test.

**Table 4:** Spearman correlations.

Pair	ρ (rho)	*p*-Value
Age vs. GradeNum	−0.031	0.782794
Age vs. pTbase	−0.179	0.028305
Age vs. AdverseCount	−0.140	0.031821
pTbase vs. AdverseCount	0.586	3.17 × 10^−16^
GradeNum vs. AdverseCount	−0.018	0.877551

ρ—Spearman rank correlation coefficient; pT—pathologic T stage; AdverseCount—number of adverse features; GradeNum—ordinal grade coding.

**Table 5:** Secondary sites.

Site	*n*	Age Mean	SD	Median	AdvT n/N	AdvT %	Node+ n/N	Node+ %	LVI n/N	LVI %	PNI n/N	PNI %	R1/R2 n/N	R1/R2%
0	57	66.8	9.9	68	14/57	24.6	4/57	7	16/57	28.1	14/57	24.6	7/14	50
Colon	47	67.6	9	68	33/47	70.2	9/47	19.1	29/47	61.7	18/47	38.3	8/29	27.6
Prostate	40	68.9	8.8	68.5	39/40	97.5	4/40	10	24/40	60	31/40	77.5	11/17	64.7
Cervix	19	60.5	9.7	62	11/19	57.9	4/19	21.1	10/19	52.6	8/19	42.1	3/6	50
Ovary	7	59.4	8.2	60	2/7	28.6	0/7	0	4/7	57.1	1/7	14.3	3/3	100
Uterus	2	80	—	80	1/2	50	0/2	0	0/2	0	0/2	0	0/0	—
Breast	1	82	—	82	0/1	0	0/1	0	0/1	0	0/1	0	0/0	—

AdvT—advanced tumor stage (pT ≥ 3); Node+—pathologic nodal metastasis present; LVI—lymph–vascular invasion; PNI—perineural invasion; R1/R2—positive margins; SD—standard deviation.

**Table 6:** Within-primary subgroup: aggressive (NEC/sarcomatoid) vs. non-aggressive.

Variable	Aggressive n/N	Aggressive %	Non-Aggressive n/N	Non-Aggressive %	*p*-Value
Advanced T (pT ≥ 3)	6/26	23.1	8/33	24.2	1
LVI	10/26	38.5	6/33	18.2	0.144927
PNI	7/26	26.9	6/33	18.2	0.540466
R1/R2 positive	1/4	25	6/10	60	0.545455
Node-positive (pN ≥ 1)	0/26	0	0/33	0.0	—
Any adverse	12/26	46.2	8/33	24.2	0.100503

Aggressive—NEC or sarcomatoid histology; NEC—neuroendocrine carcinoma; LVI—lympho–vascular invasion; PNI—perineural invasion; R1/R2—positive margins.

**Table 7:** Secondary subgroup (colon vs. prostate vs. cervix): multi-feature.

Variable	Colon n/N	Colon %	Prostate n/N	Prostate %	Cervix n/N	Cervix %	Global *p* (χ^2^)
Advanced T (pT ≥ 3)	33/47	70.2	39/40	97.5	11/19	57.9	0.000515
LVI	29/47	61.7	24/40	60	10/19	52.6	0.79047
PNI	18/47	38.3	31/40	77.5	8/19	42.1	0.000666
R1/R2 positive	8/29	27.6	11/17	64.7	3/6	50	0.044724
Any adverse	37/47	78.7	40/40	100	13/19	68.4	0.001891

LVI—lympho–vascular invasion; PNI—perineural invasion; R1/R2—positive margins; χ^2^—chi-square test; Any adverse—composite adverse endpoint.

**Table 8:** Primaries only: sarcomatoid vs. NEC vs. other primaries.

Primary Subtype	*n*	Age Median	Advanced T n/N	Advanced T %	Node+ n/N	Node+ %	LVI n/N	LVI %	PNI n/N	PNI %	R1/R2 n/N	R1/R2%	Any Adverse n/N	Any Adverse %
Sarcomatoid	15	69	6/15	40	0/15	0	5/15	33.3	5/15	33.3	3/7	42.9	7/15	46.7
NEC	11	65	2/11	18.2	0/11	0	4/11	36.4	2/11	18.2	1/2	50	4/11	36.4
Other primary	33	68	6/33	18.2	0/33	0	7/33	21.2	6/33	18.2	3/5	60	9/33	27.3

NEC—neuroendocrine carcinoma; LVI—lympho–vascular invasion; PNI—perineural invasion; R1/R2—positive margins; any adverse—composite adverse endpoint.

**Table 9:** Secondaries: colorectal (colon) vs. non-colorectal.

Group	*n*	Age Median	Advanced T n/N	Advanced T %	Node+ n/N	Node+ %	LVI n/N	LVI %	PNI n/N	PNI %	R1/R2 n/N	R1/R2%	Any Adverse n/N	Any Adverse %
Colorectal (Colon)	47	68	33/47	70.2	9/47	19.1	29/47	61.7	18/47	38.3	8/29	27.6	37/47	78.7
Non-colorectal	129	68	67/129	51.9	12/129	9.3	54/129	41.9	53/129	41.1	24/40	60	78/129	60.5

χ^2^ *p*-values: advanced T 0.046212; Node+ 0.128501; LVI 0.030599; PNI 0.873011; R1/R2 0.015498; Any adverse 0.038184; any adverse—composite adverse endpoint; LVI—lympho–vascular invasion; PNI—perineural invasion; R1/R2—positive margins.

**Table 10:** Margin cohorts (all origins): R1/R2 positive vs. R0 negative.

MarginPos.	*n*	Age Median	Primary Origin n/N	Primary Origin %	Advanced T n/N	Advanced T %	Node+ n/N	Node+ %	LVI n/N	LVI %	PNI n/N	PNI %	Any Adverse n/N	Any Adverse %
R0 (negative)	44	66	7/44	15.9	38/44	86.4	10/44	22.7	31/44	70.5	33/44	75	41/44	93.2
R1/R2 (positive)	39	62	7/39	17.9	37/39	94.9	9/39	23.1	31/39	79.5	26/39	66.7	39/39	100

χ^2^ *p*-values: Primary origin 1.000000; Advanced T 0.348113; Node+ 1.000000; LVI 0.489073; PNI 0.553035; any adverse 0.283801; R0—negative surgical margin; R1/R2—microscopic/macroscopic positive margin; LVI—lympho–vascular invasion; PNI—perineural invasion; any adverse—composite adverse endpoint.

## Data Availability

Data available on request.

## References

[1] Mohanty S.K., Lobo A., Cheng L. (2023). The 2022 revision of the World Health Organization classification of tumors of the urinary system and male genital organs: Advances and challenges. Hum. Pathol..

[2] Guo C.C., Shen S.S., Czerniak B. (2023). Recent Advances in the Classification of Bladder Cancer—Updates from the 5th Edition of the World Health Organization Classification of the Urinary and Male Genital Tumors. Bladder Cancer.

[3] Powles T., Bellmunt J., Comperat E., De Santis M., Huddart R., Loriot Y., Necchi A., Valderrama B.P., Ravaud A., Shariat S.F. (2022). Bladder cancer: ESMO Clinical Practice Guideline for diagnosis, treatment and follow-up. Ann. Oncol..

[4] PDQ Adult Treatment Editorial Board (2025). Bladder Cancer Treatment (PDQ^®^): Health Professional Version. PDQ Cancer Information Summaries.

[5] Leslie S.W., Soon-Sutton T.L., Aeddula N.R. (2025). Bladder Cancer. StatPearls.

[6] Dobruch J., Oszczudłowski M. (2021). Bladder Cancer: Current Challenges and Future Directions. Medicina.

[7] Larkins M.C., Pasli M., Bhatt A., Burke A. (2024). Squamous cell carcinoma of the bladder: Demographics and outcomes associated with surgery and radiotherapy. J. Surg. Oncol..

[8] Lagwinski N., Thomas A., Stephenson A.J., Campbell S., Hoschar A.P., El-Gabry E., Dreicer R., Hansel D.E. (2007). Squamous cell carcinoma of the bladder: A clinicopathologic analysis of 45 cases. Am. J. Surg. Pathol..

[9] Grignon D.J., Ro J.Y., Ayala A.G., Johnson D.E., Ordóñez N.G. (1991). Primary adenocarcinoma of the urinary bladder. A clinicopathologic analysis of 72 cases. Cancer.

[10] Dadhania V., Czerniak B., Guo C.C. (2015). Adenocarcinoma of the urinary bladder. Am. J. Clin. Exp. Urol..

[11] Simon N.I., Kydd A.R., Akbulut D., Takeda D., Del Rivero J., Merino M., Redd B., Lindenberg L., Mena E., Chandran E. (2025). Small cell carcinoma of the bladder: Review of pathogenesis, presentation, and management. Bladder Cancer.

[12] Li H., Parimi V., Johnson B., Kamanda S., Baraban E., Hoffman-Censits J., Kates M., McConkey D.J., Hahn N.M., Matoso A. (2025). Clinicopathologic and Prognostic Features of Sarcomatoid Urothelial Carcinoma: A Retrospective Study of 136 Patients with Emphasis on Early-Stage (pT1) Disease. Am. J. Surg. Pathol..

[13] El-Taji O., Al-Mitwalli A., Malik F., Agarwal S., Gogbashian A., Hughes R., Vasdev N., Sharma A. (2021). Secondary neoplasms of the urinary bladder-clinical management and oncological outcomes. Transl. Androl. Urol..

[14] Karaosmanoglu A.D., Onur M.R., Karcaaltincaba M., Akata D., Ozmen M.N. (2018). Secondary Tumors of the Urinary System: An Imaging Conundrum. Korean J. Radiol..

[15] Xiao G.Q., Chow J., Unger P.D. (2012). Metastatic tumors to the urinary bladder: Clinicopathologic study of 11 cases. Int. J. Surg. Pathol..

[16] Wang H.L., Lu D.W., Yerian L.M., Alsikafi N., Steinberg G., Hart J., Yang X.J. (2001). Immunohistochemical distinction between primary adenocarcinoma of the bladder and secondary colorectal adenocarcinoma. Am. J. Surg. Pathol..

[17] Roy S., Smith M.A., Cieply K.M., Acquafondata M.B., Parwani A.V. (2012). Primary bladder adenocarcinoma versus metastatic colorectal adenocarcinoma: A persisting diagnostic challenge. Diagn. Pathol..

[18] Gurel B., Ali T.Z., Montgomery E.A., Begum S., Hicks J., Goggins M., Eberhart C.G., Clark D.P., Bieberich C.J., Epstein J.I. (2010). NKX3.1 as a marker of prostatic origin in metastatic tumors. Am. J. Surg. Pathol..

[19] Yoo D., Min K.W., Pyo J.S., Kim N.Y. (2023). Diagnostic and Prognostic Roles of GATA3 Immunohistochemistry in Urothelial Carcinoma. Medicina.

[20] Suh J.W., Kim D.W., Lee J., Yang I.J., Ahn H.M., Oh H.K., Kim J.K., Lee H., Oh J.J., Lee S. (2024). Comparison of partial and total cystectomy for colorectal cancer with histologically confirmed bladder invasion. Surgery.

[21] Gaisa N.T., Hartmann A., Knüchel-Clarke R. (2023). Neue WHO-Klassifikation 2022: Harnblasenkarzinom [New WHO classification 2022: Urinary bladder cancer]. Die Pathol..

[22] Amin M.B., Greene F.L., Edge S.B., Compton C.C., Gershenwald J.E., Brookland R.K., Meyer L., Gress D.M., Byrd D.R., Winchester D.P. (2017). The Eighth Edition AJCC Cancer Staging Manual: Continuing to build a bridge from a population-based to a more “personalized” approach to cancer staging. CA Cancer J. Clin..

[23] Kondo A., Sasaki T., Kitaguchi D., Tsukada Y., Nishizawa Y., Ito M. (2019). Resection of the urinary bladder for locally advanced colorectal cancer: A retrospective comparison of partial versus total cystectomy. BMC Surg..

[24] Hamza A., Hwang M.J., Czerniak B.A., Guo C.C. (2020). Secondary tumors of the bladder: A survival outcome study. Ann. Diagn. Pathol..

[25] Chiang T.W., Chang L.W., Chiang F.F., Li J.R., Hung S.C. (2024). Colon Cancer with Bladder Invasion: A Single Center Experience. In Vivo.

[26] Zhang L.J., Wu B., Zha Z.L., Qu W., Zhao H., Yuan J., Feng Y.J. (2018). Perineural invasion as an independent predictor of biochemical recurrence in prostate cancer following radical prostatectomy or radiotherapy: A systematic review and meta-analysis. BMC Urol..

[27] Niu Y., Förster S., Muders M. (2022). The Role of Perineural Invasion in Prostate Cancer and Its Prognostic Significance. Cancers.

[28] Kraus R.D., Barsky A., Ji L., Garcia Santos P.M., Cheng N., Groshen S., Vapiwala N., Ballas L.K. (2018). The Perineural Invasion Paradox: Is Perineural Invasion an Independent Prognostic Indicator of Biochemical Recurrence Risk in Patients with pT2N0R0 Prostate Cancer? A Multi-Institutional Study. Adv. Radiat. Oncol..

[29] Lan B., Luo R., Li Y., Wang S., Jiang W., Zhong Y., Zhang X., Zheng Q., He Z., Ma B. (2025). Partial cystectomy as a surgical option for colorectal cancer patients with pathological bladder invasion: An original retrospective study. Therap. Adv. Gastroenterol..

[30] Sekar R.R., Diamantopoulos L.N., Bakaloudi D.R., Khaki A.R., Grivas P., Winters B.R., Vakar-Lopez F., Tretiakova M.S., Psutka S.P., Holt S.K. (2023). Sarcomatoid Urothelial Carcinoma Is Associated With Limited Response to Neoadjuvant Chemotherapy and Poor Oncologic Outcomes After Radical Cystectomy. Clin. Genitourin. Cancer.

[31] Chu C.E., Chen Z., Whiting K., Ostrovnaya I., Lenis A.T., Clinton T.N., Rammal R., Ozcan G.G., Akbulut D., Basar M. (2025). Clinical Outcomes, Genomic Heterogeneity, and Therapeutic Considerations Across Histologic Subtypes of Urothelial Carcinoma. Eur. Urol..

[32] Chau C., Rimmer Y., Choudhury A., Leaning D., Law A., Enting D., Lim J.H., Hafeez S., Khoo V., Huddart R. (2021). Treatment Outcomes for Small Cell Carcinoma of the Bladder: Results From a UK Patient Retrospective Cohort Study. Int. J. Radiat. Oncol. Biol. Phys..

[33] Sheng Z., Wang M., Xu Y., Xu J., Zhang C., Zhang H., Zhu J., Zeng S., Xu C., Zhang Z. (2025). Clinical features and prognosis of small cell carcinoma of the bladder: A single center retrospective analysis. Transl. Androl. Urol..

[34] Mari A., Kimura S., Foerster B., Abufaraj M., D’Andrea D., Gust K.M., Shariat S.F. (2018). A systematic review and meta-analysis of lymphovascular invasion in patients treated with radical cystectomy for bladder cancer. Urol. Oncol..

[35] Kim H., Kim M., Kwak C., Kim H.H., Ku J.H. (2014). Prognostic significance of lymphovascular invasion in radical cystectomy on patients with bladder cancer: A systematic review and meta-analysis. PLoS ONE.

[36] Leissner J., Koeppen C., Wolf H.K. (2003). Prognostic significance of vascular and perineural invasion in urothelial bladder cancer treated with radical cystectomy. J. Urol..

[37] Giannico G.A., Gown A.M., Epstein J.I., Revetta F., Bishop J.A. (2017). Role of SATB2 in distinguishing the site of origin in glandular lesions of the bladder/urinary tract. Hum. Pathol..

[38] Lin F., Shi J., Zhu S., Chen Z., Li A., Chen T., Wang H.L., Liu H. (2014). Cadherin-17 and SATB2 are sensitive and specific immunomarkers for medullary carcinoma of the large intestine. Arch. Pathol. Lab. Med..

[39] Rao Q., Williamson S.R., Lopez-Beltran A., Montironi R., Huang W., Eble J.N., Grignon D.J., Koch M.O., Idrees M.T., Emerson R.E. (2013). Distinguishing primary adenocarcinoma of the urinary bladder from secondary involvement by colorectal adenocarcinoma: Extended immunohistochemical profiles emphasizing novel markers. Mod. Pathol..

[40] Brandler T.C., Jelloul F.Z., Soto D., Das K., Rosen L., Bhuiya T.A. (2015). Young investigator challenge: Cadherin-17 and SATB2 in cytology specimens: Do these new immunostains help in differentiating metastatic colorectal adenocarcinoma from adenocarcinomas of other origins?. Cancer Cytopathol..

[41] Nakamori S., Kawai K., Dejima A., Natsume S., Ise I., Kato H., Takao M., Nakano D. (2024). Surgical outcomes of a partial or total cystectomy for colorectal cancer invasion of the bladder. Asian J. Surg..

[42] de Angelis M., Siech C., Di Bello F., Rodriguez Peñaranda N., Goyal J.A., Tian Z., Longo N., Chun F.K.H., Puliatti S., Saad F. (2025). Mortality rates in radical cystectomy patients with bladder cancer after radiation therapy for prostate cancer. BJU Int..

[43] Flammia R.S., Tufano A., Chierigo F., Würnschimmel C., Hoeh B., Sorce G., Tian Z., Anceschi U., Leonardo C., Del Giudice F. (2023). The Effect of Sex on Disease Stage and Survival after Radical Cystectomy in Non-Urothelial Variant-Histology Bladder Cancer. J. Clin. Med..

